# Therapeutic effect and autophagy regulation of myriocin in nonalcoholic steatohepatitis

**DOI:** 10.1186/s12944-019-1118-0

**Published:** 2019-10-21

**Authors:** Rui-Xu Yang, Qin Pan, Xiao-Lin Liu, Da Zhou, Feng-Zhi Xin, Ze-Hua Zhao, Rui-Nan Zhang, Jing Zeng, Liang Qiao, Chun-Xiu Hu, Guo-Wang Xu, Jian-Gao Fan

**Affiliations:** 1 Center for Fatty Liver, Department of Gastroenterology, Xinhua Hospital Affiliated to Shanghai Jiaotong University School of Medicine, Shanghai, 200092 China; 2 Storr Liver Centre, Westmead Institute for Medical Research, the University of Sydney at Westmead Hospital, Westmead, NSW 2145 Australia; 3 CAS Key Laboratory of Separation Science for Analytical Chemistry, Dalian Institute of Chemical Physics, Chinese Academy of Sciences, Dalian, 116023 China; 4 Shanghai Key Laboratory of Pediatric Gastroenterology and Nutrition, Shanghai, 200092 China

**Keywords:** Ceramides, Autophagy, High fat diet, Non-alcoholic fatty liver disease

## Abstract

**Background:**

Ceramide plays pathogenic roles in nonalcoholic fatty liver disease (NAFLD) via multiple mechanisms, and as such inhibition of ceramide de novo synthesis in the liver may be of therapeutically beneficial in patients with NAFLD. In this study, we aimed to explore whether inhibition of ceramide signaling by myriocin is beneficial in animal model of NAFLD via regulating autophagy.

**Methods:**

Sprague Dawley rats were randomly divided into three groups: standard chow (*n* = 10), high-fat diet (HFD) (*n* = 10) or HFD combined with oral administration of myriocin (0.3 mg/kg on alternate days for 8 weeks) (*n* = 10). Liver histology and autophagy function were measured. HepG2 cells were incubated with fatty acid with or without myriocin treatment. Lipid accumulation and autophagy markers in the HepG2 cells were analyzed. Serum ceramide changes were studied in 104 subjects consisting healthy adults, liver biopsy-proven patients with NAFLD and liver biopsy-proven patients with chronic hepatitis B (CHB).

**Results:**

Myriocin reversed the elevated body weight and serum transaminases and alleviated dyslipidemia in HFD fed rats. Myriocin treatment significantly attenuated liver pathology including steatosis, lobular inflammation and ballooning. By qPCR analysis, it was revealed that myriocin corrected the expression pattern of fatty acid metabolism associated genes including *Fabp1*, *Pparα*, *Cpt-1α* and *Acox-2*. Further, myriocin also restored the impaired hepatic autophagy function in rats with HFD-induced NASH, and this has been verified in HepG2 cells. Among the sphingolipid species that we screened in lipidomic profiles, significantly increased ceramide was observed in NASH patients as compared to the controls and non-NASH patients, regardless of whether or not they have active CHB.

**Conclusions:**

Ceramide may play an important regulatory role in the autophagy function in the pathogenesis of NASH. Hence, blockade of ceramide signaling by myriocin may be of therapeutically beneficial in NASH.

**Trial registration:**

Registration ID: ChiCTR-DDT-13003983. Data of registration: 13 May, 2013, retrospectively registered.

## Background

Nonalcoholic fatty liver disease (NAFLD) has become the major burden of chronic liver diseases in China over the past many years [1]. Nonalcoholic steatohepatitis (NASH) is the severe form of NAFLD and if left untreated, can evolve into end stage liver diseases such as liver cirrhosis and hepatocellular carcinoma. The classic “multiple hit” theory of NAFLD pathogenesis states that lipid accumulation initiates hepatic steatosis and subsequently triggers multiple insults, ultimately inducing NASH, cirrhosis, and hepatocellular carcinoma [2]. However, the pathogenesis of NAFLD is far more complicated, and as such more detailed understanding of the molecular pathogenesis of NAFLD particularly NASH is needed in order to develop more rational strategies for the prevention and treatment of this common liver disease.

Autophagy is a process of cellular digestion of damaged organelles and misfolded proteins in lysosomes. Autophagy could degrade cellular lipid droplets through lipophagy, which clears cellular lipid droplets and prevents the development of NAFLD [3]. Ceramides are a member of the sphingolipid family and are the key components of the phospholipid bilayer structure on the cell membranes [4]. Ceramides play important roles in multiple biological processes including cellular proliferation, differentiation, apoptosis, insulin resistance, oxidative stress and inflammation, all of which are known to be closely linked to the development of NAFLD [5–7].

Previous studies have reported that inhibition of ceramide synthesis could ameliorate NASH in rodent models through improving oxygen consumption, insulin resistance, lipoprotein metabolism and lipotoxicity [8–12]. Ceramides have been reported as effectors in autophagy regulation, and are known to mediate apoptotic pathways [13]. The role of ceramides in autophagy is controversial. For example, ceramides were reported to either promote early autophagy and apoptosis [14] or attenuate autophagy [15]. So far, very few studies have addressed the regulatory role of ceramide in autophagy during NASH development.

To elucidate the role of ceramide in the development of NASH and its regulatory role in autophagy, we evaluated the serum level of ceramide in patients with NAFLD. To explore the functional role of ceramide in autophagy, we investigated the impact of inhibiting ceramide synthesis in the high fat diet (HFD) rat models and its effect on autophagy.

## Materials and methods

### Animals and treatment

Specified pathogen free (SPF) male Sprague-Dawley rats (6 weeks old) were purchased from the Shanghai Experimental Animal Center of the Chinese Academy of Sciences and were housed in high-efficiency, particulate air-filtered cages under standard conditions of temperature, humidity, and light/dark cycle. The rats were acclimatized for 1 week after arrival with free access to water and standard chow diet prior to further experiments.

**Fig. 1:**
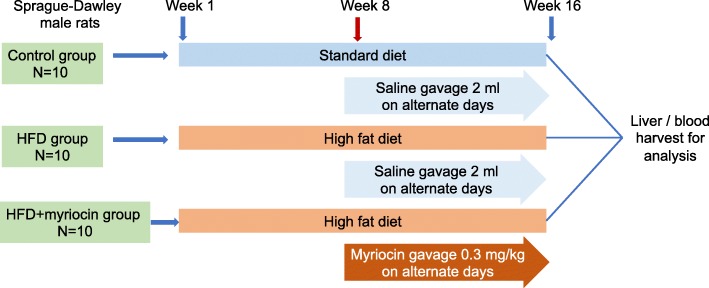
The workflow of NASH model building and myriocin intervention

Rats were randomized into three groups: Group 1 (control): rats fed with standard diet for 16 weeks (*n* = 10); Group 2 (HFD group): rats fed HFD containing 88% standard diet, 10% lard, and 2% cholesterol for 16 weeks (*n* = 10); Group 3 (intervention group): HFD + Myriocin 0.3 mg/kg on alternate days by gavage for 16 weeks (Myriocin initiated on week 8 till week 16). Detailed experimental design is shown in Fig. 1. Rats in the Groups 1 and 2 received saline by gavage as sham treatment controls. The body weight was measured and food consumption calculated on a weekly basis. All animal studies were approved by the Institutional Animal Care and Use Committee of Xinhua Hospital and the experiments conducted in accordance with the National Research Council Guide for Care and Use of Laboratory Animals.

### Blood samples and liver tissue collection for histological and lipid evaluations

At the end of 16 weeks, fasting blood samples were collected prior to animal sacrifice. The liver index and fat index were calculated from liver/body weight ratio and fat/body weight ratio. Portion of liver tissues were fixed in 4% paraformaldehyde, embedded in paraffin blocks, and further processed for histological assessment by Haematoxylin-Eosin (H&E) staining. Oil Red O (Applygen Technologies Inc., Beijing, China) staining was used to determine the extent of hepatic fat accumulation in snap-frozen sections of liver tissues. The remaining liver tissues were snap-frozen in liquid nitrogen for subsequent analysis as shown below. Intrahepatic triglycerides (TGs) were measured using a triglyceride assay kit (Nanjing Jiancheng Bioengineering Institute, Nanjing, China).

### In vitro analysis in HepG2 cells

Human hepatoblastoma cell line HepG2 was obtained from American Type Culture Collection (ATCC, Manassas, VA, USA) and cultured in Dulbecco’s modified eagle medium (DMEM) supplemented with 10% fetal bovine serum (FBS) (Gibco, CA, USA). To establish a cell model of fat overloading, free fatty acids (FFAs) consisting of palmitic acid (PA) and oleic acid (OA) (both from Sigma, St. Louis, USA) at a ratio of 1:2 were dissolved in Milli-Q water supplemented with 1% fatty acid free bovine serum albumin (Sigma, St. Louis, USA). FFAs were added into the culture medium at a final concentration of 0.5 mM, and cells were cultured for 24 h.

Cells were divided into 3 groups: Group A: naïve cells, no treatment; Group B: (Model cells): cells were treated with FFAs alone; and Group C (Treatment group): cells were treated with FFAs + myriocin. In Group A and B, the culture medium contains equal dimethylsulfoxide as treatment controls. After 24 h of treatment, Oil Red O staining was performed to detect the fat accumulation in the cells and the level of triglycerides (TG) was measured by a triglyceride assay kit, all as described above. All cellular TG levels were normalized to the protein concentrations in the same samples.

### Lipidomic analysis of human serum samples

We performed lipidomic analysis in 104 Han Chinese subjects consisting healthy controls, patients with liver biopsy-proven NAFLD and patients with active CHB who presented to Shanghai Xinhua Hospital between May 2012 and May 2014. The inclusion criteria for each group are as follows: adult people with normal blood biochemistry, negative virological markers, and normal abdominal ultrasound were included in control group (*n* = 23); patients with > 5% of their hepatocytes showing macrovesicular or mixed steatosis were included in NAFLD group (*n* = 42), and the patients therein were further divided into those with nonalcoholic fatty liver (NAFL) (*n* = 12) and those with NASH (*n* = 30) (steatosis identified in > 5% of hepatocytes, together with hepatocyte ballooning and lobular inflammation) [16], and patients with positive serum hepatitis B surface antigen (HBsAg) and/or detectable HBV DNA for at least 6 months, together with biopsy-proven active chronic hepatitis were enclosed in the CHB group (*n* = 39), which were further divided into CHB without NASH (*n* = 25) and CHB with NASH (*n* = 14) groups according to the concurrent hepatic steatosis, hepatocyte ballooning, and lobular inflammation. The exclusion criteria used were: age younger than 18 years old; excessive alcohol consumption (> 140 g per week for men and > 70 g per week for women); patients with other liver diseases including those with other types of viral hepatitis, Wilson’s disease; drug-induced liver injury; and patients with hepatocellular carcinoma.

Human blood samples were collected after overnight fasting and serum samples were separated. Sphingolipid analysis was performed as previously published [17]. In brief, total lipids were extracted by a methyl tert-butyl ether (MTBE)-based extraction. Ultra-performance liquid chromatography (UPLC) (Waters, Milford, USA) coupled with a Triple TOF 5600 mass spectrometer (AB SCIEX, USA) system was used to perform serum lipidomic analysis. Lipids separation was performed on a UPLC ACQUITY C_8_ BEN column (2.1 mm × 100 mm × 1.7 μm) (Waters, Milford, USA). The mobile phases were consisted of: phase A (60% acetonitrile in water, 10 mM ammonium acetate), and phase B (90% isopropanol, 10% acetonitrile, 10 mM ammonium acetate). The flow rate was adjusted at 0.26 ml/min, with the elution gradient as follows: started with 32% phase B was maintained in the first 1.5 min, increased to 85% B in the next 14 min, with another linear increase to 97% B from the 15.5 min to 15.6 min. This rate was maintained for the next 2.4 min, followed by 32% of B in the next 2 min for column equilibration to the next injection.

Data acquisition was performed in both positive and negative electrospray ionization (ESI) modes for full scan. The detailed parameters of mass spectrometry were as follows: ion spray voltage, 4500 V (−) and 5500 V (+); declustering potential: 100 V (−) and 100 V (+); information-dependent acquisition (IDA) with collision energy collision energy: 10 V (−) and 10 V (+); curtain gas: 35 psi; interface heater temperature: 600 °C (−) and 500 °C (+).

The study was approved by the Institutional Review Board of Xinhua Hospital. All patients provided written informed contents.

### Western blot analyses

Frozen liver tissue or cells were lysed in ice-cold radio-immunoprecipitation assay (RIPA) buffer containing phenylmethylsulfonyl fluoride (PMSF) (Beyotime, Shanghai, China). Rabbit anti-autophagosome marker microtubule-associated protein light chain-3 (LC-3) and p62 were purchased from Abcam (San Francisco, USA), and anti-actin was used as a loading control. Immune complexes were detected using a western chemiluminescent HRP substrate (Millipore Corporation, Billerica, USA).

### Real-time quantitative polymerase chain reaction

Total RNA was extracted from the liver tissue and treated cells using TRIzol (Invitrogen, Carlsbad, CA, USA). Complementary DNA was synthesized using the PrimeScript RT Reagent Kit and SYBR Premix Ex Taq (Takara, Shiga, Japan), and was subsequently used in quantitative polymerase chain reaction (qPCR). Primers for the target genes were synthesized by Sangon Biotech (Shanghai, China). The primer sequences were:

Acaca-F: GAATATCCAGATGGCCGAGA; Acaca-R: CCTTCTGCTCTGGCAAGTTC;

Pparα-F: AATGCAATCCGTTTTGGAAG; Pparα-R: TTGGCCAGAGATTTGAGGTC;

Fabp1-F: TCAAGGGGGTGTCAGAAATC; Fabp1-R: CCCAGTCATGGTCTCCAGTT;

Acox2-F: ACGTCTACGAACGCCTGTTT; Acox2-R: TGCTTCTCGGTCCCAAATCC;

Cpt-1α-F: CCACGAAGCCCTCAAACAGA; Cpt-1α-R: CACACCCACCACCACGATAA;

Fasn-F: GCCTAACACCTCTGTGCAGT; Fasn-R: GGCAATACCCGTTCCCTGAA;

Mttp-F: AGCGGCTATACAAGCTCACG; Mttp-R: GTCCACGTCCGATGAGATCC.

Relative fold-changes of gene expression were calculated using the 2^-ΔΔCt^ method.

### Statistical analysis

Two-tailed Student’s *t* test was used to compare the difference between two groups, and One-way analysis of variance with post-hoc Student-Newman-Kuels analysis was used to compare the difference among multiple groups. Mann-Whitney U test (non-normal distributed variables) and *Chi*-square test (categorical variables) were performed to compare the pairwise difference. All analyses were performed using GraphPad Prism 6.0 software (San Diego, CA, USA) and SPSS software (Version 22.0, Chicago, USA). Data are expressed as the mean ± standard error of the mean (SEM). A *p* value of < 0.05 was considered statistically significant.

## Results

### Inhibition of ceramide synthesis by myriocin attenuated HFD induced-NASH in rats

Rats fed HFD for 16 weeks demonstrated a significant increase in body weight as compared to the rats in the control group. Increased body weight in these rats was associated with widespread abnormalities in blood biochemistry, including a significant increase in the serum level of ALT, AST, TC, TG and LDL, and a significant decrease in the serum level of HDL, as compared to the control group. In comparison, treatment of rats with myriocin for 8 weeks led to a significant decrease in body weight, and this was associated with the restoration of serum transaminase levels as well as a significant improvement of dyslipidemia especially the blood cholesterol level (Table 1).

**Table 1:** Effects of myriocin on body weight and serum biochemical indicators

	Control	HFD	HFD + myriocin
Weight	566.7 ± 8.1	661.7 ± 15.9^**^	614.0 ± 14.2^#^
ALP	7.70 ± 0.68	8.40 ± 0.73	6.90 ± 0.35
ALT	37.40 ± 1.89	114.30 ± 26.71^*^	32.70 ± 3.08^#^
AST	92.10 ± 5.55	160.10 ± 16.79^**^	87.80 ± 4.02^##^
TB	1.01 ± 0.06	1.14 ± 0.22	0.78 ± 0.05
DB	0.65 ± 0.02	0.52 ± 0.05^*^	0.48 ± 0.04
HDL	0.33 ± 0.01	0.25 ± 0.01^**^	0.34 ± 0.03^#^
LDL	0.14 ± 0.01	0.42 ± 0.04^**^	0.22 ± 0.02^##^
TC	0.71 ± 0.08	1.28 ± 0.12^**^	0.81 ± 0.07^##^
TG	1.59 ± 0.05	2.36 ± 0.18^**^	2.09 ± 0.13
FPG	8.82 ± 0.39	8.51 ± 0.53	7.86 ± 0.30

Notes: Data are expressed as mean ± SEM. *: *P*<0.05, **: *P*<0.01 between HFD and control group; #: *P*<0.05, ##: *P*<0.01 between HFD and HFD + myriocin group

**Fig. 2:**
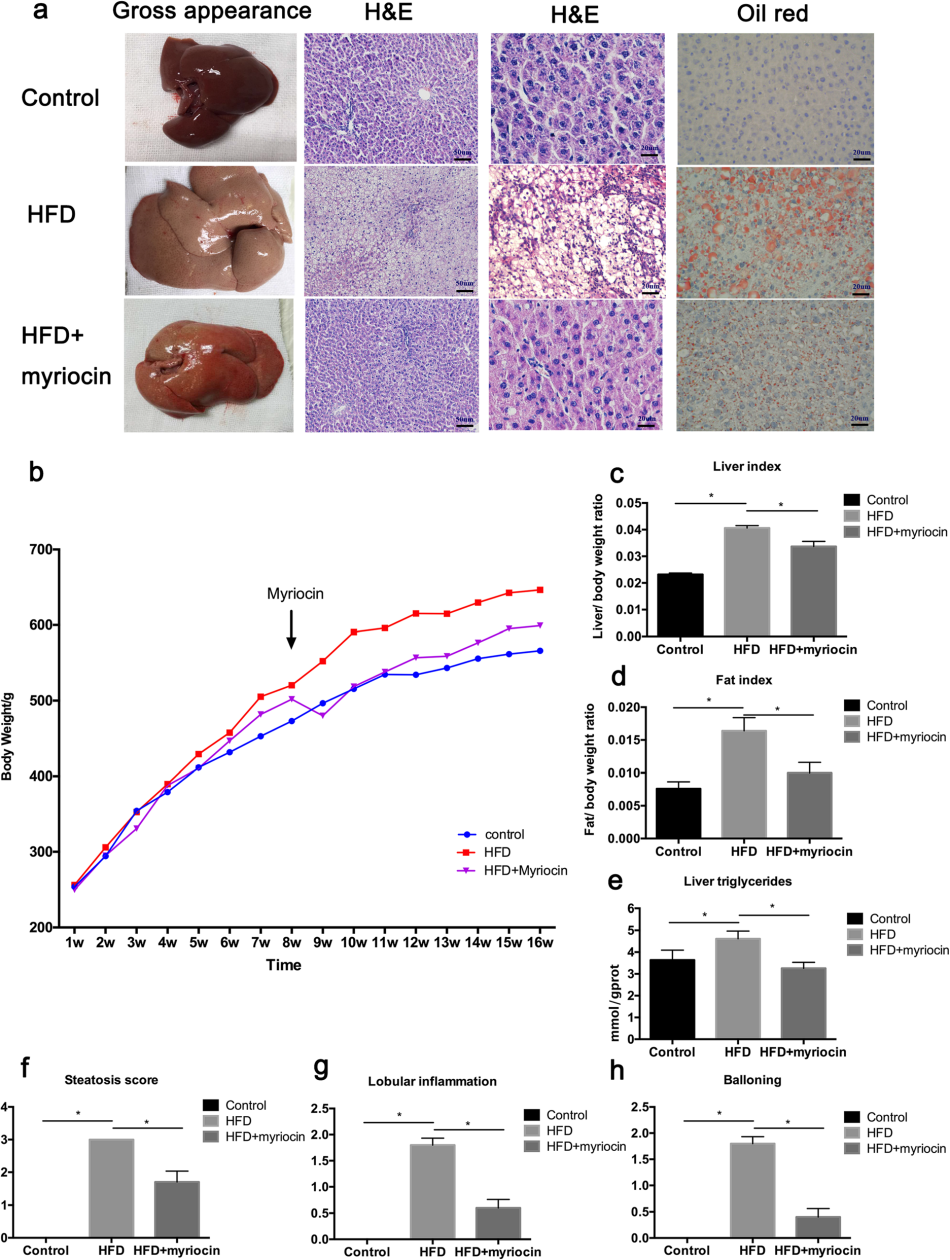
Inhibition of ceramide synthesis by myriocin alleviates NASH severity in the liver of HFD fed rats. **a** The gross appearance, H&E and Oil red O staining of liver. **b** Body weight of experimental rats; **c-d** Liver index and fat index at 16 weeks. **e** TG level in liver issues. **f-h** Histology score of steatosis, lobular inflammation and ballooning. Data represents the mean ± SEM. (*n* = 10 per group); *: *P* < 0.05

Histologically, at the end of the week 16, all rats in the HFD group developed NASH with remarkable hepatic macro-vesicular steatosis, ballooning and lobular inflammation. On H&E staining, marked lipid accumulation in the liver tissues of the HFD fed rats was seen, and this was significantly attenuated by myriocin treatment (Fig. 2a). Fat accumulation and effect of myriocin treatment were further confirmed by Oil red O staining and hepatic TG analysis (Fig. 2a, e). Treatment with myriocin also improved liver index and fat index in the HFD rats (Fig. 2c, d). These histological changes are better presented in the quantitative analyses shown in Fig. 2f-h.

### Myriocin corrected the impaired lipid metabolism in the liver of HFD rats

**Fig. 3:**
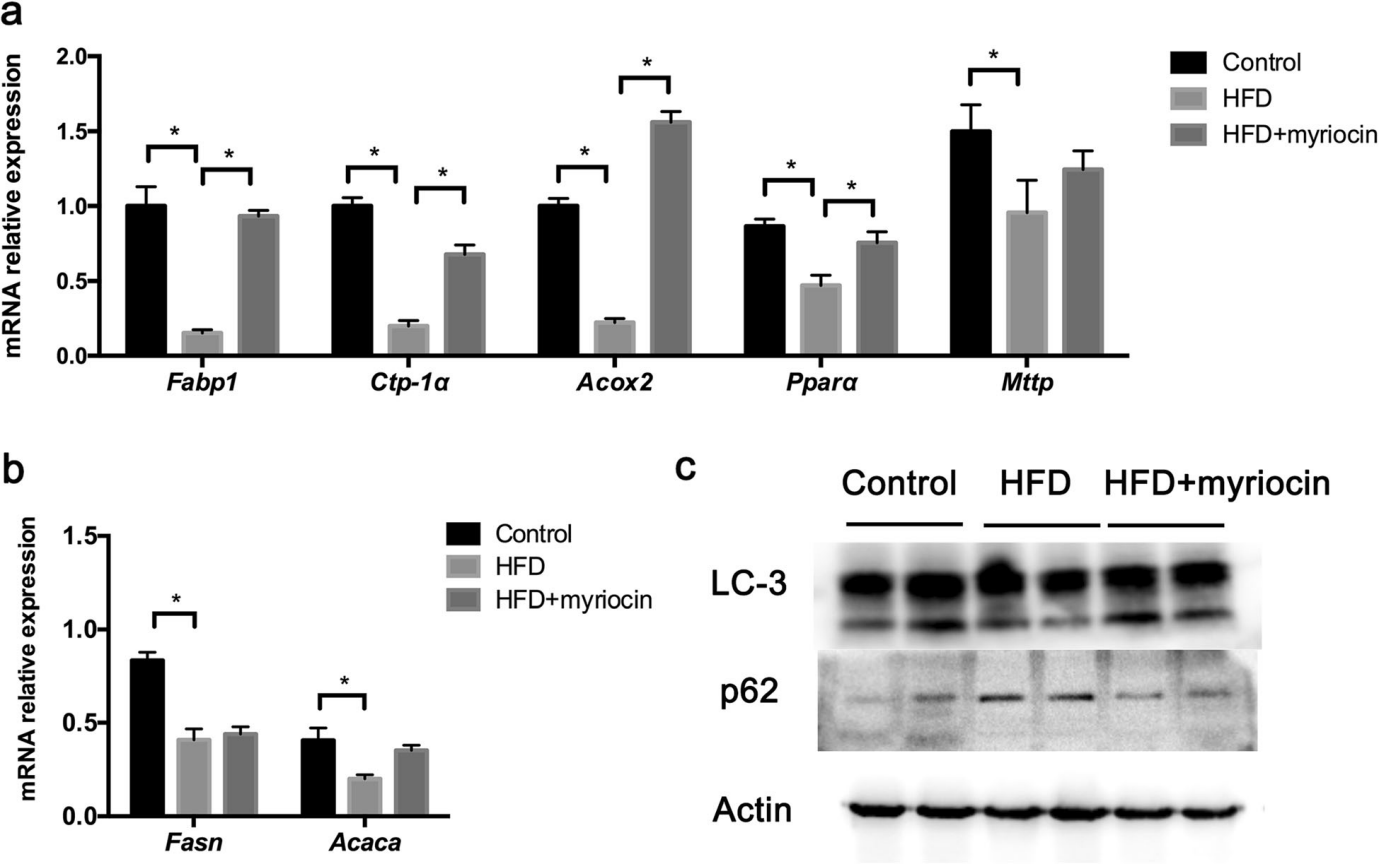
Effect of myriocin on the de novo lipogenesis, fatty acid metabolism, triglyceride transportation and autophagy in the livers of HFD fed rats. **a** Expression of genes involved in fatty acid transport and oxidation in rat livers. **b** Expression of genes involved in de novo lipogenesis in rat livers. **c** Autophagy markers (LC-3, p62) in rat livers were examined by Western blot analysis

To evaluate lipid metabolism in HFD-induced NASH, we examined the mRNA expression of genes involved in de novo lipogenesis (DNL), fatty acid oxidation (FAO) and triglyceride transportation in liver tissues. As described above, rats fed HFD developed significant steatohepatitis at 16 weeks. These rats also showed significant impairment in DNL, FAO and triglyceride transportation. Inhibition of ceramide by myriocin restored the function of fatty acid metabolism, as evidenced by increased acid binding protein 1 (*Fabp1*), peroxisomal proliferator-activated receptor α (*PPARα*), carnitine palmitoyl transferase-1α(*Cpt-1α*) and acyl CoA oxidase-2 (*Acox-2*) (Fig. 3a). However, myriocin did not significantly reverse the impaired DNL associated functions (e.g, fatty acid synthase, FASN, acetyl-CoA carboxylase, ACACA) and triglyceride transportation (e.g, microsomal triglyceride transfer protein, MTTP) (Fig. 3b).

### Myriocin corrected the impaired autophagy in the liver of HFD rats

Autophagy was reported to be involved in the development of NAFLD, we evaluated the autophagy marker, LC-3 (I, II) and p62, in the liver of HFD fed rats by Western blotting. In the HFD fed rats, impaired autophagy function was observed as indicated by significantly decreased hepatic level of LC-3II and increased p62. Following treatment with myriocin, the hepatic level of LC-3II and p62 were reversed to normal levels in these rats (Fig. 3c).

### Effect of myriocin on FFA accumulation in HepG2 cells

**Fig. 4:**
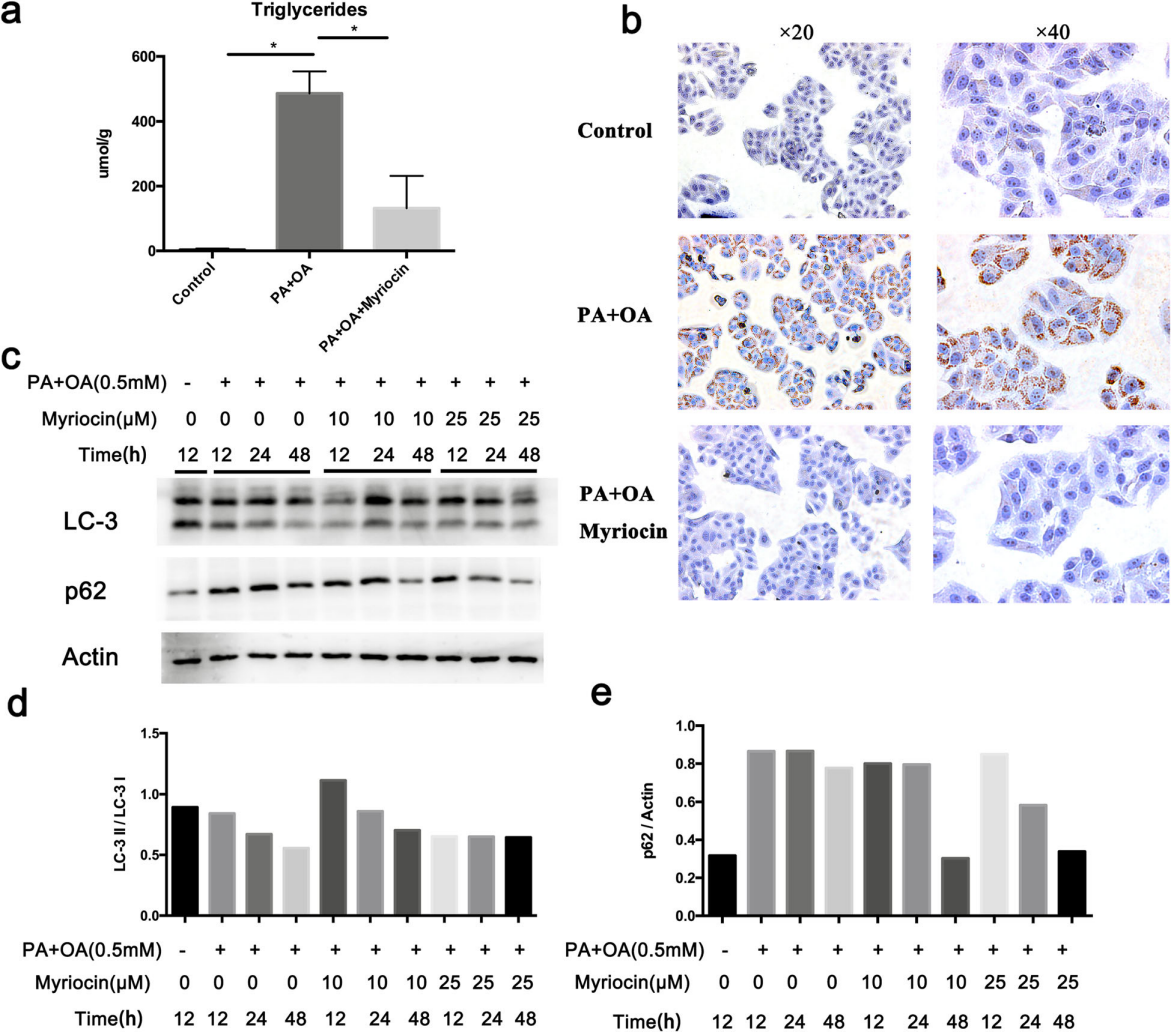
Effect of myriocin in HepG2 cells exposed to PA and OA. **a** TG content (μmol/g protein) in HepG2 cells co-cultured with PA + OA in the presence of myriocin (10 μM). **b** Oil Red O staining of HepG2 cells co-cultured with FFAs and myriocin (10 μM). **c** Expression of autophagy markers (LC-3, p62) was examined by Western blot in HepG2 cells co-cultured with FFAs and various concentrations of myriocin (0, 10, and 25 μM). **d-e** Quantitative analysis of LC-3 II/I ratio and p62/Actin ratio in **c**

To simulate the effect of myriocin on lipid accumulation in hepatocytes, we performed in vitro studies using HepG2 cells treated with fatty acids as a model. By Oil Red O staining, marked lipid accumulation was observed in HepG2 cells treated with FFAs for 24 h, which was alleviated by myriocin (10 μM) (Fig. 4b). As expected, myriocin significantly reduced the cellular TG levels induced by the FFAs (Fig. 4a).

### Effect of myriocin on the autophagy markers in FFA-treated HepG2 cells

HepG2 cells were co-incubated with 0.5 mM FFAs in the presence or absence of 10 μM or 25 μM myriocin for 12, 24 and 48 h, and the expression level of autophagy markers LC-3 (I, II) and p62 was determined by western blotting. Decreased LC-3 II/I ratio and increased p62 was observed at 12, 24 and 48 h post FFAs incubation, indicating autophagy suppression by FFAs. Treatment of cells with myriocin (10 μM) restored the level of LC-3 II/I to normal at 12 and 24 h, and reduced the level of p62 at 48 h. However, it should be noted that a higher dose of myriocin (25 μM) did not alter the level of LC-3 II/I but decreased p62 (Fig. 4c-e).

### Significantly increased ceramide level in the serum samples of NASH patients

**Fig. 5:**
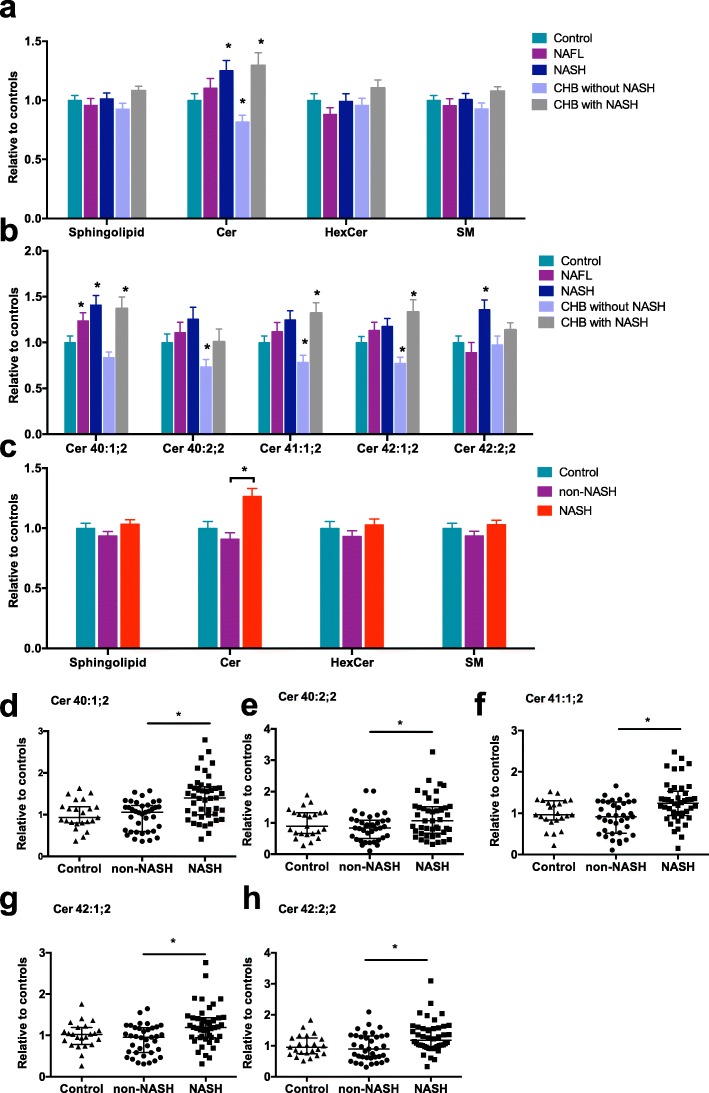
Ceramides were significantly elevated in NASH subjects. **a** Serum sphingolipids in normal subjects and patients with NAFL, NASH, CHB with or without NASH. **b** Analysis of specific ceramide species in five subgroups. **c** analysis of serum sphingolipids in control, non-NASH subjects (NAFL and CHB without NASH) and NASH subjects (NASH and CHB with NASH). **d-h** Comparison of specific ceramide species between the non-NASH subjects (NAFL and CHB without NASH) and the NASH subjects (NASH and CHB with NASH)

The general characteristics of the 104 subjects involved in this study are shown in Table 2. No significant difference in the gender and age was seen between five different subgroups. Compared to the control group, elevated liver enzymes and liver stiffness were found in patients in the NAFL, NASH, and CHB groups (regardless of NASH status). However, patients in the NASH subgroup and CHB with NASH subgroups were more obese, with higher controlled attenuation parameters (CAP) and significant dyslipidemia. A total of 31 sphingolipid species including five ceramides (Cer), three hexosylceramides (HexCer) and 23 sphingomyelins (SM) were identified in this patient cohort. Serum sphingolipid analysis is shown in Fig. 5a. Analysis of specific ceramide specie in five subgroups showed that most of the ceramides were elevated in the NASH subgroup and in the CHB with NASH subgroup (Fig. 5b). Overall, NASH subjects (NASH or CHB with NASH, *n* = 44) showed a more significant elevation in the total ceramides and individual specific ceramide species as compared to the non-NASH subjects (NAFL, and CHB without NASH, *n* = 37), (Fig. 5c-h).

**Table 2:** General characteristics of the human study population

Subgroup	Control	NAFL	NASH	CHB without NASH	CHB with NASH
n	23	12	30	25	14
Age	39.91 ± 1.19	42.33 ± 3.57	38.20 ± 2.66	36.76 ± 2.59	39.00 ± 3.75
Male (%)	14 (60.87%)	8 (66.67%)	18 (60.00%)	16 (64.00%)	9 (64.29%)
Waistline	80.96 ± 1.32	89.17 ± 1.19**	92.34 ± 1.43**	80.38 ± 3.28	90.00 ± 1.68**
BMI	23.80 ± 0.52	26.74 ± 0.85**	27.73 ± 0.61**	22.61 ± 0.64	26.25 ± 0.53**
TB	15.63 ± 0.79	13.48 ± 1.46	19.06 ± 3.32	25.30 ± 5.00	12.98 ± 0.85
DB	2.53 ± 0.19	4.86 ± 0.69**	9.43 ± 3.03*	11.15 ± 3.63*	3.97 ± 0.40**
ALP	69.30 ± 3.87	78.98 ± 9.89	95.38 ± 7.60**	84.73 ± 8.66	96.01 ± 15.22*
γ-GT	25.47 ± 4.51	31.76 ± 4.22	157.26 ± 56.40*	82.35 ± 20.69*	46.39 ± 9.02
ALT	24.17 ± 2.23	43.94 ± 5.70**	99.55 ± 11.87**	113.91 ± 30.18**	59.13 ± 9.79**
AST	21.17 ± 0.93	24.13 ± 1.54	60.32 ± 6.20**	60.93 ± 12.75**	41.73 ± 7.23**
TC	4.60 ± 0.18	4.38 ± 0.26	4.84 ± 0.19	3.75 ± 0.17**	5.43 ± 0.30*
TG	1.37 ± 0.16	1.51 ± 0.24	2.05 ± 0.22*	1.25 ± 0.15	2.70 ± 0.83*
HDL	1.25 ± 0.04	1.30 ± 0.07	1.17 ± 0.06	1.12 ± 0.06	1.31 ± 0.08
LDL	2.83 ± 0.15	2.78 ± 0.09	2.89 ± 0.13	2.32 ± 0.12*	2.93 ± 0.22
FPG	5.00 ± 0.14	6.02 ± 0.57*	5.67 ± 0.30	4.83 ± 0.21	6.31 ± 0.84
Stiffness	4.35 ± 0.20	6.68 ± 0.97*	12.20 ± 2.50**	10.13 ± 1.44**	7.64 ± 0.93**
CAP	222.83 ± 7.98	280.42 ± 10.56**	314.97 ± 9.27**	241.43 ± 12.84	274.93 ± 12.38**

Notes: Data are expressed as mean ± SEM

*:*P* < 0.05, **: *P* <0.01 vs control group

## Discussion

Our in vitro and in vivo data clearly indicate that blockade of ceramide signaling by myriocin could alleviate NASH severity, and improve the serum transaminases, triglycerides, cholesterol, liver pathology and fatty acid metabolism. In addition, myriocin could also reverse the impaired autophagy function, indicating an important regulatory role of ceramides in autophagy function in the pathogenesis of NASH. Importantly, the regulatory role of ceramides in the pathogenesis of human NASH is supported by the data we obtained from the lipidomic studies in 104 human subjects, where patients with NASH showed a significant elevation of ceramides.

Ceramides are critical components of sphingolipids with multiple biological functions [18]. Aberrant ceramide signaling has been linked to obesity, insulin resistance and metabolic disorders [19, 20]. Previous studies had reported that saturated fatty acids from de novo lipogenesis and/or from diets could induce insulin resistance via stimulating ceramide synthesis and increasing hepatic influx of fatty acids [21]. Ceramide has been reported to inhibit insulin receptor substrate 1 (IRS1), phosphatidylinositol 3-kinase (PI-3 K) and Akt/PKB, all of which play essential roles in insulin signaling pathway [22]. In addition to its key role in regulating insulin resistance, ceramide could also inhibit FAO [23], promote triglyceride synthesis, induce hepatic lipid accumulation and lipotoxicity, induce oxidative stress, stimulate cytokine expression, induce mitochondrial dysfunction and provoke apoptosis, all of these are mechanistically involved in the pathogenesis of NASH [5, 21, 24–27]. To support our findings, previous studies in biopsy-proven NAFLD patients had reported that insulin resistance in the human liver is associated with increased ceramide concentrations through de novo synthesis [24]. Six different ceramide synthases (CerS) that differ in tissue distribution and substrate specificity account for the diversity in acyl-chain composition of distinct ceramide species [23]. CerS6 related palmitoyl ceramide (C16:0 ceramide) could negatively regulate insulin signaling through inhibiting FAO [28, 29]. CerS2 is the dominant hepatic CerS isoform and it preferentially makes very long-chain ceramides (C22:0, C24:0, C24:1). Hence, in the liver CerS2 is far more abundant than any other CerS isoforms. In the present study, we screened the sphingolipid profile including ceramides, hexosylceramides and sphingomyelins, and observed a significant elevation of ceramide in the NASH subjects regardless of whether they had active HBV infection. It was noted that in our study, the increased serum ceramides are mainly very long chain ceramides, indicating liver may be a potential source of ceramide synthesis.

Ceramides are mainly derived through three pathways: the de novo pathway, the sphingomyelinase pathway and the salvage pathway. The de novo synthesis of ceramide takes place in endoplasmic reticulum and is enhanced by inflammation or triggered by excess of saturated fatty acids [30]. The de novo synthesis of ceramide starts with serine and palmitoyl CoA by the action of palmitoyltransferase (SPT), the rate-limiting enzyme of ceramide biosynthesis [23]. Myriocin is a potent inhibitor of SPT. Inhibition of ceramide synthesis by myriocin has been reported to improve oxygen consumption, insulin resistance and lipotoxicity, and ameliorate NASH in vivo [8–12]. In our study, HFD induced NASH in all animals fed HFD for 16 weeks. Blockade of ceramide signaling by myriocin for 8 weeks could significantly improve HFD-induced NASH as evidenced by improved obesity, dyslipidemia and histologic changes. The impact of myriocin in improving dyslipidemia in rats was recapitulated in cultured HepG2 cells exposed to fatty acids.

Ceramides were reported to trigger redox signaling and activate reactive oxygen species (ROS) generating enzymes in NASH [26, 31, 32]. In our study, we found that ceramide inhibition could restore the expression of genes associated with fatty acid oxidation that was previously impaired due to ROS injury and mitochondrial dysfunction in NASH. However, blockade of ceramide signaling by myriocin had no significant effect on the liver DNL associating genes. Therefore, the therapeutic roles of myriocin in hepatic steatosis are likely through regulation of mitochondrial function and oxidative stress, but not through inhibition of DNL. Our study also suggests a deleterious role of ceramide in lipid accumulation and in fatty acid oxidation, thereby acting as a harmful mediator in the pathogenesis of NASH.

One of the important findings of the current study is that autophagy dysfunction appears to play an important role in NASH. Autophagy is a process of cellular digestion of damaged organelles and misfolded proteins in lysosomes. Autophagy degrades the cellular lipid droplets through a process called lipophagy, which is essential in eliminating hepatocellular lipid droplets, and hence in preventing NAFLD development [3]. Upregulated autophagy activity has been reported in the development of NAFLD [33]. In our present study, significantly impaired autophagy function was observed in HFD-induced NASH in rats. We speculated that there was a dynamic change of autophagic function in the development of NAFLD, ranging from autophagy upregulation at the early stage to the decompensated autophagic function at the late stage of NASH development. So far, few studies have addressed the role of impaired autophagy function and its regulation by ceramide in NASH patients. Limited publications on the role of ceramides in regulating autophagy have provided controversial results in that ceramide was reported to promote early autophagy and apoptosis in one study [13] while in another study, ceramide was shown to attenuate autophagy [15]. In fact, ceramide has been shown to mediate two opposing autophagic pathways, being regulating cell survival or cell death, a state referred to as “autophagy paradox”, though the exact mechanism of ceramide regulating the autophagy paradox remains unclear [14]. In our study, we found that inhibition of ceramide could restore the impaired autophagy in NASH rats, and this was verified in HepG2 cells. We speculated that upregulation of autophagy mediated by ceramide inhibition was either due to the direct regulation of the autophagy by ceramide itself or by the overall improvement of inflammation and of oxidative stress.

It must be noted that our study has some limitations. Firstly, in the human studies, only serum sphingolipids but not the intrahepatic sphingolipids were measured; secondly, the underlying mechanisms of how ceramide intervention affects autophagy in NASH rats and cellular model were inadequately studied. Therefore, further mechanistic studies are needed.

## Conclusions

Ceramide may play an important regulatory role in the autophagy function in the pathogenesis of NASH. Hence, blockade of ceramide signaling may be of therapeutically beneficial in NASH patients. Further studies are warranted to elucidate the mechanisms by which ceramide regulates autophagy during the pathogenesis of NAFLD.

## Data Availability

All data related to this study are available from the corresponding author (Professor JG Fan) upon request.

## Abbreviations

ALP: Alkaline phosphatase
ALT: Alanine aminotransferase
AST: Aspartate aminotransferase
BMI: Body mass index
CAP: Controlled attenuation parameter
CHB: Chronic hepatitis B
DB: Direct bilirubin
DNL: De novo lipogenesis
FAO: Fatty acid oxidation
FPG: Fasting plasma glucose
HDL: High density lipoprotein
HFD: High-fat diet
LDL: Low density lipoprotein
NAFL: Nonalcoholic fatty liver
NAFLD: Nonalcoholic fatty liver disease
NASH: Nonalcoholic steatohepatitis
TB: Total bilirubin
TC: Total cholesterol
TG: triglycerides
γ-GT: gamma-glutamyltransferase
